# The Duration of an Exposure Response Gradient between Incident Obstructive Airways Disease and Work at the World Trade Center Site: 2001-2011

**DOI:** 10.1371/currents.dis.8a93e7682624698558a76a1fa8c5893f

**Published:** 2015-05-20

**Authors:** Charles B. Hall, Xiaoxue Liu, Rachel Zeig-Owens, Mayris P. Webber, Thomas K. Aldrich, Jessica Weakley, Theresa Schwartz, Hillel W. Cohen, Michelle S. Glaser, Brianne L. Olivieri, Michael D. Weiden, Anna Nolan, Kerry J. Kelly, David J. Prezant

**Affiliations:** Department of Epidemiology and Population Health and Saul B. Korey Department of Neurology, Albert Einstein College of Medicine, Bronx, New York, USA; Montefiore Medical Center, Bronx, New York, USA; Fire Department of the City of New York, Brooklyn, New York, USA; Montefiore Medical Center, Bronx, New York, USA; Fire Department of the City of New York, Brooklyn, New York, USA; Department of Epidemiology and Population Health, Montefiore Medical Center and Albert Einstein College of Medicine, Bronx, New York, USA; Fire Department of the City of New York, Brooklyn, New York, USA; Division of Pulmonary Medicine, Department of Medicine, Montefiore Medical Center and Albert Einstein College of Medicine, Bronx, New York, USA; Montefiore Medical Center, Bronx, New York, USA; Fire Department of the City of New York, Brooklyn, New York, USA; Montefiore Medical Center, Bronx, New York, USA; Fire Department of the City of New York, Brooklyn, New York, USA; Department of Epidemiology and Population Health, Albert Einstein College of Medicine, Bronx, New York, USA; Montefiore Medical Center, Bronx, New York, USA; Fire Department of the City of New York, Brooklyn, New York, USA; Montefiore Medical Center, Bronx, New York, USA; The Fire Department of the City of New York, Brooklyn, New York, USA; Division of Pulmonary, Critical Care and Sleep Medicine, Department of Medicine, New York University School of Medicine, New York, New York, USA; The Fire Department of the City of New York, Brooklyn, New York, USA; Division of Pulmonary, Critical Care and Sleep Medicine, Department of Medicine, New York University School of Medicine, New York, New York, USA; The Fire Department of the City of New York, Brooklyn, New York, USA; The Fire Department of the City of New York, Brooklyn, New York, USA; The Fire Department of the City of New York, Brooklyn, New York, USA; Albert Einstein College of Medicine and Montefiore Medical Center, Bronx, New York, USA

## Abstract

**Background**: Adverse respiratory effects of World Trade Center (WTC) exposure have been widely documented, but the length of time that exposure remains associated with disease is uncertain. We estimate the incidence of new cases of physician-diagnosed obstructive airway disease (OAD) as a function of time since 9/11/2001 in WTC-exposed firefighters.

**Methods:** Exposure was categorized by first WTC arrival time: high (9/11/2001 AM); moderate (9/11/2001 PM or 9/12/2001); or low (9/13-24/2001). We modeled relative rates (RR) and 95% confidence intervals (CI) of OAD incidence by exposure over the first 10 years post-9/11/2001, estimating the time(s) of change in the RR with change point models. We further examined the relationship between self-reported lower respiratory symptoms and physician diagnoses.

**Results:** Change points were observed at 15 and 84 months post-9/11/2001, with relative incidence rates for the high versus low exposure group of 4.02 (95% CI 2.62-6.16) prior to 15 months, 1.90 (95% CI 1.49-2.44) from months 16 to 84, and 1.20 (95% CI 0.92-1.56) thereafter. Incidence in all exposure groups increased after the WTC health program began to offer free coverage of OAD medications in month 63. Self-reported lower respiratory symptoms in the first 15 months had 80.6% sensitivity, but only 35.9% specificity, for eventual OAD diagnoses.

**Conclusions**: New OAD diagnoses are associated with WTC exposure for at least seven years. Some portion of the extended duration of that association may be due to delayed diagnoses. Nevertheless, our results support recognizing OAD among rescue workers as WTC-related even when diagnosed years after exposure.

## Background

The inhalation of chemicals, particulate matter (dusts and fibers), and the incomplete products of combustion during occupational and environmental disasters have long been associated with respiratory disorders.^1^ While there is substantial literature 2 ^,^ 3 ^,^ 4 ^,^ 5 ^,^ 6 ^,^ 7 ^,^ 8 ^,^ 9 ^,^ 10 ^,^ 11 on the association between respiratory diseases and chronic environmental exposures such as air pollution, and long-term occupational exposure in mining, silica handling, and construction, and other industries, much remains to be learned regarding the biological mechanisms that cause such disease and the presumed latency period between acute exposure and disease onset.

The destruction of the World Trade Center (WTC) buildings after a terrorist attack on September 11, 2001, resulted in a massive, intense dust cloud that was found to contain a huge variety of irritants including partially combusted and/or pulverized wood, paper, and jet fuel; pulverized construction materials including asbestos, glass, silica, fiberglass, and concrete; complex organic chemicals; lead; and other metals.^12^ Increased incidence of respiratory disease has been reported in firefighters who worked in the rescue/recovery effort and in other worker and non-worker cohorts.^13^^,^ 14 ^,^ 15 ^,^ 16 ^,^ 17 Obstructive airway disease (OAD), such as asthma and chronic bronchitis, have been shown to be associated with intensity of exposure as measured by arrival time at the WTC site.^18^ New-onset OAD continues to be observed many years after WTC exposure,^19^ contrary to conventional wisdom that irritant-induced asthma is triggered within a relatively short time after exposure.^20^ We set out to determine whether late-onset OAD demonstrated an exposure intensity gradient similar to early-onset disease, which would be consistent with WTC-causation, or whether the exposure gradient disappears over time.

## Methods

**Data Sources**

Demographic information was obtained from the Fire Department of the City of New York (FDNY) employee database. The FDNY medical program, run by the FDNY Bureau of Health Services (FDNY-BHS), has used an electronic medical record with ICD-9 coded diagnoses since 1996. Physician diagnoses for this study were obtained from this electronic medical record. Since October 2001, FDNY-BHS also has collected data from self-administered health questionnaires completed during routine monitoring exams conducted every 12-18 months. We used questionnaire information to categorize WTC exposure intensity, smoking status, and the presence of lower respiratory symptoms of cough, shortness of breath, or wheeze.

**Cohort**

The study population consisted of uniformed male FDNY firefighters who were on active duty as of 9/11/2001 and had participated in the WTC rescue/recovery effort on or before 9/24/2001. We excluded those who did not consent for research (1.6%), those with evidence of pre-9/11 OAD based on FDNY-BHS medical records (5.1%), those who did not have a visit with an FDNY-BHS physician prior to 9/11/2011 (1.3%), and those with an unknown smoking history (n=3) (Figure 1). The final analysis cohort consisted of 9,778 individuals.

**Flowchart of exclusions from analysis cohort.**
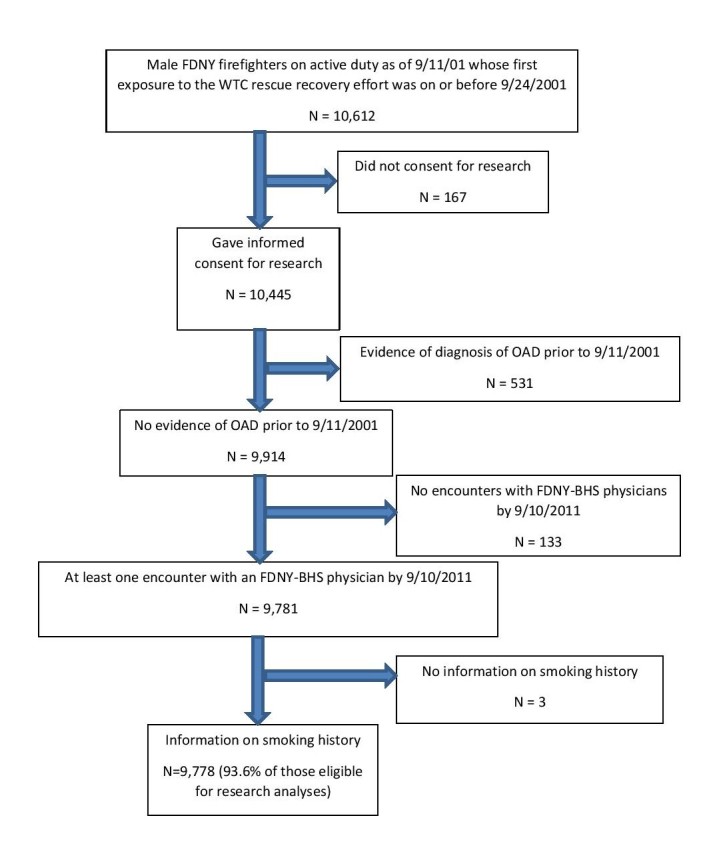

**Exposure measure**

The measure of exposure intensity was based on the arrival time at the WTC site as self-reported on their first medical monitoring questionnaire. Arrival the morning of 9/11 was defined as high exposure for this cohort, arrival the afternoon of 9/11 or any time on 9/12/2001 was defined as moderate exposure, and arrival any time from 9/13/2001 to 9/24/2001 was defined as low exposure. Persons who did not participate in the rescue/recovery effort prior to 9/25/2001 were excluded from the analysis.

**Smoking Status**

Smoking history was characterized by the last known smoking status as reported on the medical monitoring questionnaires. Participants were considered as “ever” smokers if they reported smoking at any time, and as “never” smokers if they consistently reported never smoking on all questionnaires. As previously noted, three persons whose smoking status was consistently missing were excluded from the final analytic cohort.

**Outcome measure**

The primary outcome was an OAD diagnosis by an FDNY-BHS physician. OAD diagnoses included diagnoses of asthma, chronic bronchitis, or chronic obstructive pulmonary disease (COPD)/emphysema. To avoid misclassification of persons with transient symptoms as cases, we required that asthma or COPD/emphysema diagnoses be recorded in the FDNY-BHS medical records on at least two occasions, at least 30 days apart. For chronic bronchitis, we required that two diagnoses occur within one year of each other, followed by at least one additional chronic bronchitis diagnosis during a three year period beginning one year after the date of initial diagnosis. The earliest date of each series of diagnoses was selected as the date of incident OAD. If an individual had a diagnosis of more than one OAD subtype, the first subtype to be diagnosed was used for the analyses. Twelve individuals had more than one “first diagnosis” on the same day and were assigned the OAD subtype of asthma. For the analyses of the relationship between lower respiratory symptoms and physician diagnosis, the primary outcome was the self-report of a lower respiratory symptom of cough, wheeze, or shortness of breath on the medical monitoring questionnaires described above.

**Statistical methods**

Details of the methodological approach have been reported previously.^21^ Briefly, we used piecewise exponential survival models with change points to estimate relative rates across the three exposure groups during follow up (9/11/2001 to 9/10/2011). Piecewise exponential survival models are similar to Cox regression models, but with baseline hazards that are allowed to change at a fixed number of time intervals rather than with every new event. We allowed the baseline hazard to change every three months over the follow up period. The change points are the times that the relative rates change (increase or decrease) during the follow-up period; these change points are estimated from the data using profile likelihood.^21^^,^ 22 ^,^ 23 A change point after which relative incidences did not differ significantly (p<0.05) from one would show that the exposure-response relationship between WTC exposure intensity and incident OAD was limited to the period prior to the change point. All models included as covariates: age on 9/11/2001, retirement status, if applicable, as a time-dependent predictor, and self-reported smoking status (ever vs. never) as of the last completed questionnaire. Seasonality was taken into account through the varying of baseline rates every three months. In sensitivity analyses we estimated the relative rates for OAD subtypes of asthma and of non-asthma (chronic bronchitis and COPD/emphysema). SAS version 9.4 (SAS Institute, Cary, NC, USA, http://www.sas.com) was used for the analyses.

**Details of statistical model equations**

What follows are the equations for statistical models that compare three exposure groups (low, moderate, high). Low exposure intensity is assumed to be the reference group. These are all piecewise exponential survival models with change points included to model changing relative risks over time. The models are similar to ones used in a previous report.

A null model, assuming constant relative rates of incidence over the entire follow-up period, contains no change points, can be expressed as follows:

\begin{equation*}\log\left( Y_{ik} \right)= \log\left( T_{ik} \right)+\Sigma_{k=1}^{k=n}a_{k}w_{ik}+\beta_{1}x_{i1}+\beta_{1}x_{i2} +\Sigma _{l}\gamma _{l} z _{il}  \end{equation*}

Here, *Y_ik_* is the number of incident cases of disease, modeled to follow a Poisson distribution given the covariates, and *T_ik_* the total person time at risk for a particular strata corresponding to time interval *k* and the exposures indicated by the values of *x_i1_*, *x_i2_*, and the *z_il_'s* For a ten year follow-up there are *n=40* three month time intervals and the *α_k _'s* represent the log of the baseline incidence, i.e. the incidence for the low exposure group for individuals all of whose *z_ij_ 's* take the value zero, with the *w_ik _'s* as dummy variables that indicate the time interval for the stratum. These correspond to the baseline hazard in a Cox regression model but have true incidence interpretation in this fully parametric approach. *x_i1_* and *x_i2_* are dummy exposure variables, with *x*_*i1*_taking the value *1* for moderate exposure and *0* otherwise, and *x_i2_* taking the value *1* for high exposure and zero otherwise; *β_1_* is thus the log relative hazard between the moderate and low exposure groups, and β_2_ the log relative hazard between the high and low exposure groups. These relative hazards have true incidence rate ratio interpretation in this fully parametric modeling approach. The *z_il_ 's* are the additional covariates include in the model and the *γ_l_ 's* are the log relative hazard for the additional covariates. A Poisson likelihood, mathematically equivalent to that of the exponential survival model, was used for the model fit and as the goodness of fit measure.

In the non-null model, change points are introduced to allow the relative hazards between exposure groups to vary over the follow-up time, as this expression for a model with* p* distinct nontrivial change points shows as follows (equation 2):

\begin{equation*}\log(Y_{ik})=\log(T_{ik})+\Sigma _{k=1}^{k=n}\alpha_{k} \omega_{ik}+\Sigma _{j=1}^{p=1}  \beta _{1j}x_{i1}1(\tau_{j-1}<t_{ik}\leq \tau _j)\end{equation*}

\begin{equation*}+\Sigma _{j=1}^{p+1} \beta _{2j}x_{i2}1(\tau x_{j-1}<t_{ik}\leq \tau _{j})+\Sigma_{l}\Gamma_{l}z_{il}\end{equation*}

Here, *β_1j_* and* β*_2j_ are the log relative hazards (moderate vs. low exposure and high vs. low exposure, respectively), for the* j^th^* period of follow-up, defined as between the change points at time *τ_j-1_* and *τ_j_* , with *τ*_*0*_ *=0 *and *τ_p+1_=120* months from 9/11/2001, with the time variable *t_ik_* defined similarly, and *1(-)* is the dummy variable function taking the value *1* when the argument is true and *0* otherwise. For *p>0,* **τ_1_,** **τ_2,...,_ τ_p _**would be nontrivial change points. For *p=0* this reduces to model (1). Models with different numbers of change points are compared using likelihood ratio tests. A significantly better fit to a model with one or more change points indicates that the exposure-response relationship changes over time. If after some time *τ_j_*, neither* β_1,j+1_* and* β_2,j+1_* are significantly different from zero, that would show that the exposure-response relationship between WTC exposure and incident OAD is limited to the first *τ*_*j*_ months of follow-up.

**Institutional review board**

The study was approved by the Institutional Review Board of Montefiore Medical Center and the Albert Einstein College of Medicine. All study participants provided written informed consent.

## Results

Table 1 describes the cohort by exposure intensity. 16.3% were in the high exposure group, 71.5% in the moderate exposure group, and 12.2% in the low exposure group. The three exposure groups were similar in age, other demographics, and smoking history. There was a clear exposure-response gradient across the groups, with higher levels of exposure associated with higher rates of OAD.

**Table 1. Characteristics of FDNY Firefighter Population.** New York City, New York, 9/11/2001-9/10/2011.

	High Exposure(N=1,589)	Moderate Exposure (N=6,992)	Low Exposure (N=1,197)	Total (N=9,778)
Age on 9/11, years, median (IQR)	39.8 (34.7-45.2)	39.3 (34.3-44.8)	40.9 (35.2-46.2)	39.6 (34.5-45.0)
Non-Hispanic White, N (%)	1455 (91.6)	6617 (94.6)	1111 (92.8)	9183 (93.9)
Black or African-American, N (%)	63 (4.0)	145 (2.1)	37 (3.1)	245 (2.5)
Hispanic, N (%)	61 (3.8)	212 (3.0)	48 (4.0)	321 (3.3)
Current smoker as of last questionnaire, N (%)	91 (5.7)	376 (5.4)	83 (6.9)	550 (5.6)
Former smoker as of last questionnaire, N (%)	492 (31.0)	2,211 (31.6)	389 (32.5)	3,092 (31.6)
Retire during study, N (%)	488 (30.7)	2,122 (30.4)	449 (37.5)	3,059 (31.3)
Physician visits, median (IQR)	27 (12-46)	28 (13-47)	26 (11-46)	28 (13-47)
Mean physician visits per year (95% CI)	4.1 (2.2-6.7)	3.8 (2.0-6.0)	3.5 (1.6-5.8)	3.8 (2.0-6.1)
OAD, N (%)	466 (29.3)	1,613 (23.1)	207 (17.3)	2,286 (23.4)
Asthma, N (%)	275 (17.3)	902 (12.9)	112 (9.4)	1,289 (13.2)
Non-Asthma OAD, N (%)	191 (12)	711 (10.2)	95 (7.9)	997 (10.2)
Overall OAD incidence per 100 person-years	3.70	2.72	2.02	2.78
Total person-time, months	145,943.33	685,610.48	118,837.19	950,391.00
Person-time, months, mean (SD)	91.8 (38.2)	98.1 (33.1)	99.3 (32.5)	97.2 (34.0)

The rate of visits to FDNY-BHS physicians differed by exposure group only in the first year after 9/11/2001. After the first year, rates gradually declined throughout the follow-up period as the acute effects of WTC exposure diminished (Figure 2). This is important because, on average, the opportunity to be diagnosed with OAD by an FDNY-BHS physician did not vary by exposure group after the first year of follow-up.

**Average annual number of encounters with FDNY-BHS physicians since 9/11/2001:**
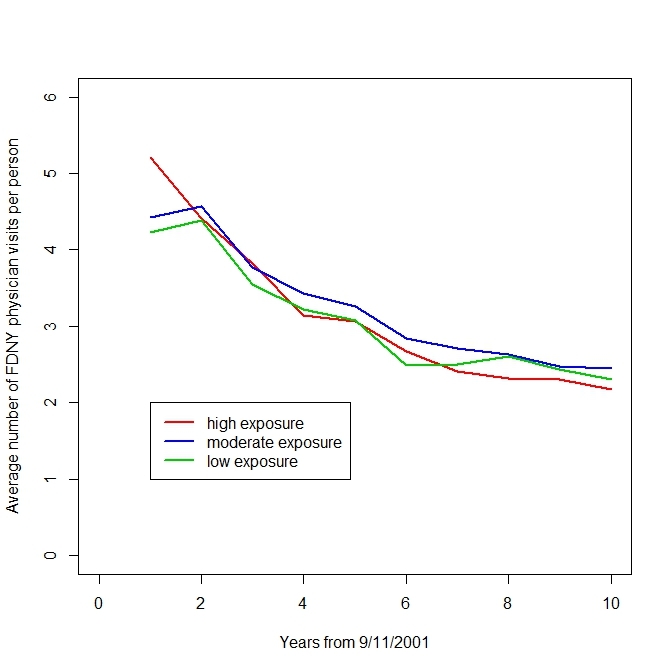

Analyses showed that a model in which relative rates of OAD changed twice during the 10 year study period best fit the data, with change points at 15 and 84 months post-9/11 in the best fitting model. A model with two change points fit significantly better than a model with a one change point (likelihood ratio test p=0.042), but adding a third change point did not improve the fit over the 2 change point model (likelihood ratio test p=0.24). Table 2 shows the relative rates for the three periods identified by the change points; the effect of exposure on incident physician-diagnosed OAD was strong in the first 15 months, smaller for months 16 through 84, and no longer statistically significant for months 85 through 120. Similar results were observed in models restricting the outcome to asthma, or to non-asthma OAD subtypes.

**Table 2. Incidence rate ratios (hazard ratios) for incidence of physician-diagnosed obstructive airway disease (OAD) as a function of exposure intensity for the three periods identified as the times of rate ratio change that best fit the data.** The analysis controls for age on 9/11, retirement status (time dependent), smoking status (ever vs. never), and implicitly for seasonality (by quarter).

Period	Exposure Contrast	Any OAD Rate Ratio (95% CI)	Asthma Rate Ratio (95% CI)	Non-Asthma OAD Rate Ratio (95% CI)
	High vs. Low	4.02 (2.62,6.16)	4.47 (2.48, 8.06)	3.56 (1.91, 6.66)
	High vs. Moderate	2.11 (1.71,2.61)	2.05 (1.56, 2.70)	2.21 (1.60, 3.07)
	Moderate vs. Low	1.90 (1.26,2.86)	2.18 (1.24, 3.84)	1.61 (0.89, 2.92)
15-84 months	High vs. Low	1.90 (1.49,2.44)	2.02 (1.47, 2.77)	1.74 (1.17, 2.59)
	High vs. Moderate	1.20 (1.03,1.39)	1.28 (1.06, 1.56)	1.06 (0.83, 1.36)
	Moderate vs. Low	1.59 (1.28,1.98)	1.57 (1.19, 2.08)	1.64 (1.16, 2.32)
85-120 months	High vs. Low	1.20 (0.92,1.56)	1.36 (0.93,1.99)	1.03 (0.71, 1.51)
	High vs. Moderate	1.17 (0.97,1.42)	1.21 (0.92,1.58)	1.14 (0.86, 1.51)
	Moderate vs. Low	1.02 (0.82,1.27)	1.13 (0.82,1.55)	0.91 (0.67,1.23)

Figure 3 shows the estimated incidence of new physician-diagnosed OAD by quarter for each of the exposure groups. The incidence rates for the low exposure group correspond to the values of a baseline hazard function in a Cox regression model. The incidence graphs show the large exposure effects early after 9/11/2001, and continued elevated incidence in the high and moderate exposure groups through month 84 compared to the low exposure group. Seasonal effects are clearly apparent, with higher incidence in winter months. The increase in physician-diagnosed OAD after the WTC medication program took effect in 2007 (year six) is seen in all exposure groups. Retirement status was weakly associated with OAD diagnosis, with retired persons having non-significantly lower rates of OAD (incidence rate ratio 0.89, 95% CI [0.79-1.00]). Surprisingly, smoking was not associated with OAD diagnoses (incidence rate ratio 1.03 for ever smokers vs. never smokers, 95% CI [0.94-1.12]).

**Incidence of physician-diagnosed OAD in WTC firefighters exposed to the WTC rescue/recovery effort since 9/11/2001 and by exposure intensity.**
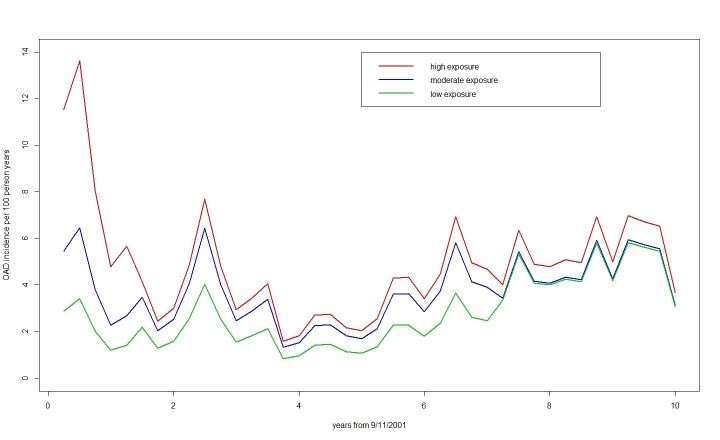

**The relationship between symptoms and diagnoses**

We examined how lower respiratory symptoms were associated with OAD diagnoses within individuals. Table 3 shows the relationship of physician diagnoses of OAD to prior self-reported OAD symptoms of cough, wheeze, and shortness of breath. Most persons who developed OAD reported symptoms prior to physician diagnosis, and this was true throughout the follow-up period. However, the majority of persons who never received an OAD diagnosis also reported OAD symptoms. Of the 2,286 persons who received an OAD diagnosis during the follow-up period, 1,934 (84.6%) took a medical monitoring questionnaire and completed the question on self-reported lower respiratory symptoms during the first 15 months. Of those, 1,559 reported at least one lower respiratory symptom during the first 15 months, for an overall sensitivity of 80.6%.

**Table 3. Fraction of persons reporting OAD symptoms on medical monitoring questionnaires by month of OAD diagnosis.** *1,471 persons did not complete a medical monitoring questionnaire during the first 15 months and are excluded from this column.

Month of OAD diagnosis	#/N (%) self-reporting OAD symptoms in first 15 months*	#/N self-reporting OAD symptoms in any prior period
1-15	321/351 (91.5%)	-
16-60	477/585 (81.5%)	477/685 (69.6%)
61-84	288/369 (78.1%)	319/432 (73.8%)
85-120	473/629 (75.2%)	554/745 (74.4%)
Never	4,088/6,373 (64.2%)	4,682/7,492 (62.5%)
Entire cohort	5,647/8,307 (68.0%)	9,778

Persons whose first physician diagnosis of OAD was long after 9/11/2001 were very likely to have reported lower respiratory symptoms shortly after 9/11. 6,373 persons did not receive a diagnosis during the follow-up period and responded to the medical monitoring questionnaire item for lower respiratory symptoms; of those, 4,088 reported at least one lower respiratory symptom, for a specificity of 35.9%. Only 189 of 954 individuals (19.8%) who were diagnosed with OAD between months 16 and 84 and who completed the questionnaire during the first 15 months did not report any lower respiratory symptoms at that time.

The majority of those who reported lower respiratory symptoms shortly after 9/11/2001 did not receive an OAD diagnosis during the study period. 1,559 of 5,647 individuals reporting a lower respiratory symptom during the first 15 months received a physician diagnosis of OAD during follow-up, for a positive predictive value of 27.6%.

## Discussion

We found that for the first seven years after 9/11/2001, earlier arrival time at the WTC site was associated with higher rates of physician diagnoses of incident OAD. This finding is consistent with crude analyses and also with other modeling approaches not reported here. These results support recognizing OAD among rescue workers as WTC-related even if diagnosed long after the exposure.

We cannot determine, however, how much of the apparent lag in WTC-associated diagnoses represents the progressive development of disease compared with the possibility of delayed diagnoses of OAD for two main reasons. First, in 2007, free medications for the treatment of OAD began to be provided to FDNY WTC-exposed firefighters. This program change required that diagnoses be given by FDNY physicians, and was followed by large increases in new incident physician diagnoses of OAD for the next two years in all three exposure groups. Nonetheless, the effect of WTC exposure remained, even as the number of incident physician diagnoses increased.

Second, most persons receiving an OAD diagnosis reported lower respiratory symptoms early in the follow-up period even when the diagnosis was made years later. For some, it may have taken time for symptoms to become sufficiently severe and chronic to warrant a diagnosis. Indeed 19.8% of those responding to questionnaire items on lower respiratory symptoms during the first 15 months post-9/11 who received an OAD diagnosis between month 16 and month 84 failed to report any symptoms on that early questionnaire.

But it is also likely that some cases could have been diagnosed earlier. We believe that this is particularly likely to be true for cases diagnosed after year five, as the program change resulted in higher incidence rates starting in the sixth year when free OAD medications became available to those diagnosed by FDNY-BHS physicians. In addition, it is likely that symptoms reported on confidential medical monitoring questionnaires preceded the FDNY-BHS physician diagnosis for some FDNY members who may have felt their symptoms to be of insufficient severity to warrant further testing, medications, or disability retirement. Similarly, some diagnoses may have been made by outside physicians at some time prior to diagnosis by FDNY-BHS physicians.

Thus we believe that the length of time between WTC exposure and OAD diagnoses must be interpreted as a combination of disease natural history and the characteristics of the health system that produce those diagnoses. Biologically, the natural history of this cohort suggests that a brief exposure to irritants predisposes a subgroup of susceptible individuals to non-resolving pulmonary inflammation producing the signs and symptoms supporting the diagnosis of OAD years after the exposure. Trained immunity involving epigenetic reprogramming of myeloid cells is one mechanism whereby a brief challenge can produce prolonged increased inflammation upon re-challenge.^24^

A study limitation is that we did not have access to a demographically similar cohort of non-WTC exposed individuals. Therefore, we can neither support nor rule out an association of WTC exposure with OAD that is not intensity-related. Nor can we assess the possible duration of such an association; we cannot rule out the possibility that WTC-exposed individuals in general remain at higher risk for incident OAD beyond the study period. An additional limitation is that we did not have access to the records of private physicians who might have given diagnoses of OAD that were unknown to FDNY-BHS, or were unknown prior to the 2007 change in medication coverage. However, we believe that it is likely that some of the early symptoms represented acute irritation that did not progress to OAD which is, by definition, a chronic disease. Further research is needed to understand why individuals with similar exposures experience vastly different disease progression; i.e., why some with acute symptoms progress to chronic disease while others appear to fully recover. Exposures to the FDNY firefighter responders, even for those in what is defined as the “low” exposure group in this study, are likely to have been more intense than exposures of other rescue/recovery workers so the results here may not be generalizable to other rescue/recovery cohorts. Nevertheless, the study has many strengths, including low rates of loss to follow-up, consistent diagnostic criteria applied throughout the follow-up period, and an exposure measure that has been validated in other studies.

Our results support recognizing OAD among WTC rescue workers as WTC-related even if diagnosed long after the exposure. Hopefully, the lessons learned from the study of the health effects of the WTC-responders can contribute to greater protections and better care for the survivors and responders in future disasters.

## Competing Interests

This research was supported by National Institute of Occupational Safety and Health Cooperative Agreement # U01 OH010412 (PI: Charles B. Hall).

Additional support came from National Institute of Occupational Safety and Health Contracts 200-2011-39378 (PI: David J. Prezant), 200-2001-39383 (PI: Kerry J. Kelly); Cooperative Agreements U10-OH008242 (PI: David J. Prezant) and U10-OH008243 (PI: Kerry J. Kelly).

Dr. Hall, Ms. Liu, Ms. Weakley, Ms. Schwartz, and Dr. Webber receive additional salary support from National Institute of Occupational Safety and Health Cooperative Agreement U01 OH010711.

Drs. Weiden and Nolan receive salary support from National Institute of Occupational Safety and Health Cooperative Agreement U01 OH010726 (PI: Michael P. Weiden).

Dr. Nolan receives salary support from National Center for Advancing Translational Sciences grant UL1 TR000038 (PI: Bruce N. Cronstein).

Dr. Aldrich and Dr. Hall received salary support from National Institute of Occupational Safety and Health Cooperative Agreement U01 OH010411 (PI: Thomas K. Aldrich).

The authors have no additional competing interests to declare.

## References

[1] Prezant DJ, Levin S, Kelly KJ, Aldrich TK. Upper and lower respiratory diseases after occupational and environmental disasters. Mt Sinai J Med. 2008 Mar-Apr;75(2):89-100. PubMed PMID:18500710. 18500710 10.1002/msj.20028

[2] Thurston GD, Bates DV. Air pollution as an underappreciated cause of asthma symptoms. JAMA. 2003 Oct 8;290(14):1915-7. PubMed PMID:14532321. 14532321 10.1001/jama.290.14.1915

[3] De Leon SF, Thurston GD, Ito K. Contribution of respiratory disease to nonrespiratory mortality associations with air pollution. Am J Respir Crit Care Med. 2003 Apr 15;167(8):1117-23. PubMed PMID:12684250. 12684250 10.1164/rccm.200205-409OC

[4] Chatkin JM, Tarlo SM, Liss G, Banks D, Broder I. The outcome of asthma related to workplace irritant exposures: a comparison of irritant-induced asthma and irritant aggravation of asthma. Chest. 1999 Dec;116(6):1780-5. PubMed PMID:10593805. 10593805 10.1378/chest.116.6.1780

[5] Kogevinas M, Zock JP, Jarvis D, Kromhout H, Lillienberg L, Plana E, Radon K, Torén K, Alliksoo A, Benke G, Blanc PD, Dahlman-Hoglund A, D'Errico A, Héry M, Kennedy S, Kunzli N, Leynaert B, Mirabelli MC, Muniozguren N, Norbäck D, Olivieri M, Payo F, Villani S, van Sprundel M, Urrutia I, Wieslander G, Sunyer J, Antó JM. Exposure to substances in the workplace and new-onset asthma: an international prospective population-based study (ECRHS-II). Lancet. 2007 Jul 28;370(9584):336-41. PubMed PMID:17662882. 17662882 10.1016/S0140-6736(07)61164-7

[6] Santos MS, Jung H, Peyrovi J, Lou W, Liss GM, Tarlo SM. Occupational asthma and work-exacerbated asthma: factors associated with time to diagnostic steps. Chest. 2007 Jun;131(6):1768-75. PubMed PMID:17505048. 17505048 10.1378/chest.06-2487

[7] Petran M, Cocârlă A, Băiescu M. Association between bronchial hyper-reactivity and exposure to silicon carbide. Occup Med (Lond). 2000 Feb;50(2):103-6. PubMed PMID:10829429. 10829429 10.1093/occmed/50.2.103

[8] Malo JL, L'archevêque J, Castellanos L, Lavoie K, Ghezzo H, Maghni K. Long-term outcomes of acute irritant-induced asthma. Am J Respir Crit Care Med. 2009 May 15;179(10):923-8. PubMed PMID:19234102. 19234102 10.1164/rccm.200810-1550OC

[9] Tarlo SM, Broder I. Irritant-induced occupational asthma. Chest. 1989 Aug;96(2):297-300. PubMed PMID:2666043. 2666043 10.1378/chest.96.2.297

[10] Malo JL, Chan-Yeung M, Bernstein DI. Asthma in the Workplace. 4th ed. Boca Raton, FL: CRC Press; 2013. DOI: 10.3109/9781841849256

[11] Malo JL, Ghezzo H, D'Aquino C, L'Archevêque J, Cartier A, Chan-Yeung M. Natural history of occupational asthma: relevance of type of agent and other factors in the rate of development of symptoms in affected subjects. J Allergy Clin Immunol. 1992 Dec;90(6 Pt 1):937-44. PubMed PMID:1460199. 1460199 10.1016/0091-6749(92)90466-f

[12] Lioy PJ, Weisel CP, Millette JR, Eisenreich S, Vallero D, Offenberg J, Buckley B, Turpin B, Zhong M, Cohen MD, Prophete C, Yang I, Stiles R, Chee G, Johnson W, Porcja R, Alimokhtari S, Hale RC, Weschler C, Chen LC. Characterization of the dust/smoke aerosol that settled east of the World Trade Center (WTC) in lower Manhattan after the collapse of the WTC 11 September 2001. Environ Health Perspect. 2002 Jul;110(7):703-14. PubMed PMID:12117648. 12117648 10.1289/ehp.02110703 PMC1240917

[13] Banauch GI, Dhala A, Prezant DJ. Pulmonary disease in rescue workers at the World Trade Center site. Curr Opin Pulm Med. 2005 Mar;11(2):160-8. PubMed PMID:15699790. 15699790 10.1097/01.mcp.0000151716.96241.0a

[14] Farfel M, DiGrande L, Brackbill R, Prann A, Cone J, Friedman S, Walker DJ, Pezeshki G, Thomas P, Galea S, Williamson D, Frieden TR, Thorpe L. An overview of 9/11 experiences and respiratory and mental health conditions among World Trade Center Health Registry enrollees. J Urban Health. 2008 Nov;85(6):880-909. PubMed PMID:18785012. 18785012 10.1007/s11524-008-9317-4 PMC2587652

[15] Ekenga CC, Friedman-Jiménez G. Epidemiology of respiratory health outcomes among World Trade Center disaster workers: review of the literature 10 years after the September 11, 2001 terrorist attacks. Disaster Med Public Health Prep. 2011 Sep;5 Suppl 2:S189-96. PubMed PMID:21908698. 21908698 10.1001/dmp.2011.58

[16] Kim H, Herbert R, Landrigan P, Markowitz SB, Moline JM, Savitz DA, Todd AC, Udasin IG, Wisnivesky JP. Increased rates of asthma among World Trade Center disaster responders. Am J Ind Med. 2012 Jan;55(1):44-53. PubMed PMID:22068920. 22068920 10.1002/ajim.21025

[17] Weakley J, Webber MP, Ye F, Zeig-Owens R, Cohen HW, Hall CB, Kelly K, Prezant DJ. Agreement between obstructive airways disease diagnoses from self-report questionnaires and medical records. Prev Med. 2013 Jul;57(1):38-42. PubMed PMID:23597657. 23597657 10.1016/j.ypmed.2013.04.001

[18] Webber MP, Glaser MS, Weakley J, Soo J, Ye F, Zeig-Owens R, Weiden MD, Nolan A, Aldrich TK, Kelly K, Prezant D. Physician-diagnosed respiratory conditions and mental health symptoms 7-9 years following the World Trade Center disaster. Am J Ind Med. 2011 Sep;54(9):661-71. PubMed PMID:21966080. 21966080 10.1002/ajim.20993 PMC3181470

[19] Weakley J, Webber MP, Gustave J, Kelly K, Cohen HW, Hall CB, Prezant DJ. Trends in respiratory diagnoses and symptoms of firefighters exposed to the World Trade Center disaster: 2005-2010. Prev Med. 2011 Dec;53(6):364-9. PubMed PMID:21930151. 21930151 10.1016/j.ypmed.2011.09.001

[20] Santos MS, Jung H, Peyrovi J, Lou W, Liss GM, Tarlo SM. Occupational asthma and work-exacerbated asthma: factors associated with time to diagnostic steps. Chest. 2007 Jun;131(6):1768-75. PubMed PMID:17505048. 17505048 10.1378/chest.06-2487

[21] Glaser MS, Webber MP, Zeig-Owens R, Weakley J, Liu X, Ye F, Cohen HW, Aldrich TK, Kelly KJ, Nolan A, Weiden MD, Prezant DJ, Hall CB. Estimating the time interval between exposure to the World Trade Center disaster and incident diagnoses of obstructive airway disease. Am J Epidemiol. 2014 Aug 1;180(3):272-9. PubMed PMID:24980522. 24980522 10.1093/aje/kwu137 PMC4108044

[22] Hall CB, Lipton RB, Sliwinski M, Stewart WF. A change point model for estimating the onset of cognitive decline in preclinical Alzheimer's disease. Stat Med. 2000 Jun 15-30;19(11-12):1555-66. PubMed PMID:10844718. 10844718 10.1002/(sici)1097-0258(20000615/30)19:11/12<1555::aid-sim445>3.0.co;2-3

[23] Hall CB, Ying J, Kuo L, Lipton RB. Bayesian and profile likelihood change point methods for modeling cognitive function over time. Computational Statistics & Data Analysis. 2003 February; 42(1-2):91-109. DOI: 10.1016/S0167-9473(02)00148-2

[24] Cheng SC, Quintin J, Cramer RA, Shepardson KM, Saeed S, Kumar V, Giamarellos-Bourboulis EJ, Martens JH, Rao NA, Aghajanirefah A, Manjeri GR, Li Y, Ifrim DC, Arts RJ, van der Veer BM, Deen PM, Logie C, O'Neill LA, Willems P, van de Veerdonk FL, van der Meer JW, Ng A, Joosten LA, Wijmenga C, Stunnenberg HG, Xavier RJ, Netea MG. mTOR- and HIF-1α-mediated aerobic glycolysis as metabolic basis for trained immunity. Science. 2014 Sep 26;345(6204):1250684. PubMed PMID:25258083. 25258083 10.1126/science.1250684 PMC4226238

